# Evidence of Rapid Modulation by Social Information of Subjective, Physiological, and Neural Responses to Emotional Expressions

**DOI:** 10.3389/fnbeh.2017.00231

**Published:** 2018-01-09

**Authors:** Martial Mermillod, Delphine Grynberg, Léo Pio-Lopez, Magdalena Rychlowska, Brice Beffara, Sylvain Harquel, Nicolas Vermeulen, Paula M. Niedenthal, Frédéric Dutheil, Sylvie Droit-Volet

**Affiliations:** ^1^Univ. Grenoble Alpes, CNRS, LPNC, Grenoble, France; ^2^Institut Universitaire de France, Paris, France; ^3^Univ. Lille, CNRS, CHU Lille, UMR 9193 - SCALab - Sciences Cognitives et Sciences Affectives, Lille, France; ^4^Univ. Lille, Villeneuve d'Ascq, France; ^5^Université Clermont Auvergne, CNRS, LaPSCo, Clermont-Ferrand, France; ^6^Queen's University Belfast, Belfast, United Kingdom; ^7^IPSY, Université Catholique de Louvain, Louvain-la-Neuve, Belgium; ^8^Fund for Scientific Research (FRS-FNRS), Brussels, Belgium; ^9^Department of Psychology, University of Wisconsin-Madison, Madison, WI, United States; ^10^Université Clermont Auvergne, Centre National de la Recherche Scientifique, LaPSCo, Stress Physiologique et Psychosocial, CHU Clermont-Ferrand, Santé Travail Environnement, WittyFit, Clermont-Ferrand, France; ^11^Faculty of Health, School of Exercise Science, Australian Catholic University, Melbourne, VIC, Australia

**Keywords:** emotion, social cognition, electromyography, electroencephalography, top-down processes, embodiment theory

## Abstract

Recent research suggests that conceptual or emotional factors could influence the perceptual processing of stimuli. In this article, we aimed to evaluate the effect of social information (positive, negative, or no information related to the character of the target) on subjective (perceived and felt valence and arousal), physiological (facial mimicry) as well as on neural (P100 and N170) responses to dynamic emotional facial expressions (EFE) that varied from neutral to one of the six basic emotions. Across three studies, the results showed reduced ratings of valence and arousal of EFE associated with incongruent social information (Study 1), increased electromyographical responses (Study 2), and significant modulation of P100 and N170 components (Study 3) when EFE were associated with social (positive and negative) information (vs. no information). These studies revealed that positive or negative social information reduces subjective responses to incongruent EFE and produces a similar neural and physiological boost of the early perceptual processing of EFE irrespective of their congruency. In conclusion, the article suggests that the presence of positive or negative social context modulates early physiological and neural activity preceding subsequent behavior.

## Introduction

Extant behavioral research suggests the relevance of top-down processes in the use of concepts (Schyns et al., 1998; Vermeulen et al., 2009), percepts (Bar, 2004; Quétard et al., 2015, 2016), and affects (Scherer, 1997; Hess et al., 2007; Rudrauf et al., 2008; Niedenthal et al., 2009). For instance, participants' personality (e.g., Campanella et al., 2012) or facial expressions (Niedenthal et al., 2009) modulate the detection or recognition of emotional facial expressions (EFE). In addition, research has pointed to a modulation of EFE processing by social factors, such as the degree of friendship between the participant and the target (Hess et al., 1995) or the social norms of the participants (Scherer, 1997).

In a social neuroscience perspective, recent studies show that the physiological (i.e., facial mimicry) and neural underpinnings of the perception of EFE may also depend upon high-level social factors associated with the targets or with the participants. For instance, facial emotions expressed by cultural in-group (compared to out-group) targets modulate facial mimicry of participants (Mondillon et al., 2007) and event-related potentials in response to these EFE (Kubota and Ito, 2007). In another study, happy expressions of targets presented as future interaction partners (socially relevant) lead to enhanced late positive potentials (LPP) amplitudes relative to non socially-relevant targets (Bublatzky et al., 2014). Because LPP amplitudes are larger for high arousing (vs. low arousing) stimuli (Schupp et al., 2006), these results suggest that social relevance may function as top-down factor on positive EFE, leading observers to perceive happy faces as more relevant (i.e., larger LPP amplitude).

Together, these studies suggest that social factors associated with the participant, the target or with the social interaction between them may influence the processing of EFE at subjective (recognition accuracy), physiological (facial mimicry), and neural (event-related potentials) levels. However, most of these studies have either focused on the participants' characteristics (e.g., Likowski et al., 2008) or manipulated the social appraisal of the targets by varying either the perceptual features (e.g., ethnicity) of the face itself (e.g., Kubota and Ito, 2007) or the nature of the interaction between the participant and the target (e.g., Bublatzky et al., 2014). There is so far little evidence that rapid physiological and neural responses to EFE are influenced by higher-order social information about the expresser. This theoretical question is even more acute if this expresser is strictly identical at a perceptual level (i.e., the same actor presented with a positive social valence for half of the participants and a negative social valence for the other half). To our knowledge, only one study investigated whether positive, negative or neutral characteristics of the target modulated facial mimicry of EFE (Likowski et al., 2008). This article revealed that positive traits (e.g., kind) enhanced Zygomatic activity for happy expressions and Corrugator activity for sad expressions. Moreover, negative (e.g., malicious) and neutral traits had no influence on Zygomatic but reduced Corrugator activity for sad expressions. Although this study highlights the influence of positive or negative traits on EMG responses to EFE (i.e., enhanced mimicry for positive targets), each avatar had their specific traits thus preventing the control for the perceptual stability of facial expressions.

Therefore, the aim of the present article was to examine whether high-level social information about the same perceptual target influence subjective responses to EFE but also impact automatic facial mimicry and neural processes involved in EFE categorization. The investigation of such influence is highly relevant as recent models propose that the psychological and neural underpinnings of the perception of EFE depend on the understanding of social context (Niedenthal et al., 2010). Furthermore, we postulate that the effect of social information on EFE processing could be interpreted within the theoretical framework of emotional embodied simulation (Dimberg, 1990; Hess et al., 1995; Barsalou, 1999; Niedenthal, 2007), which assumes that the processing of emotional information is grounded in the brain's perceptual, affective, and sensory-motor systems. Several studies have shown that the processing of emotional concepts (e.g., emotional words or EFE) leads individuals to simulate this concept and re-create the corresponding bodily experience (Niedenthal et al., 2009). Recent research also suggests that imitation of perceived and simulated emotions plays a causal role in the recognition of facial expressions (Künecke et al., 2014). Indeed, preventing participants from simulating emotional concepts (i.e., blocking participants' facial expressions) during EFE recognition task reduced recognition accuracy (Havas et al., 2010). Finally, it has been shown that the recognition of EFE is influenced by the activation (or inhibition) of facial muscles used to express the perceived emotion (Niedenthal, 2007). Similar findings were reported with direct Transcranial Magnetic Stimulation (TMS) modulation of the sensorimotor cortex (Pitcher et al., 2008). Together, these studies support that EFE are bodily grounded and that their processing is modulated through the embodied simulation. With respect to the moderating role of social information on the effect of EFE on subjective, physiological, and neural responses, we postulate that through their influence on embodied simulation of EFE, social factors associated with the target may influence their processing.

However, a recurrent methodological limitation of articles related to top-down processes was that the stimuli differ in terms of emotion but also in terms of perceptual properties. For instance, it is difficult to determine to what extent the EEG activity observed by Achaibou et al. (2008) at the level of the P100 and N170 components for, as an example, happy expression, is due to the feeling of happiness or, alternatively, to the big white area (related to the teeth) inherent to happy facial expressions. In other words, the limitations of this paper relate to the fact that the stimuli differed not only in terms of emotions but also in terms of perceptual properties that could have induced the EEG difference reported. Therefore, we have decided to address this methodological concern while extending their princeps results to more general social information. In a new set of experiments, we used the same dynamic videos of facial expressions as used in Achaibou et al. (2008). However, before each stimulus, we provided participants with a social information (either positively- or negatively-valenced, compared to a control situation without social information) about the actor displaying the emotional expression. We then recorded participants behavioral, EEG, and EMG responses to these stimuli.

Therefore, the present article aimed to investigate and extend the top-down influence of social information during perception of EFE on their subjective, physiological, and neural responses. This is based on previous findings showing that mimicry, assessed by electromyography (EMG) activity, but also P100 and N170 event-related potentials (ERP), were modulated during a EFE recognition task of dynamic stimuli (Achaibou et al., 2008). In other words, the aim of the present research was to examine whether the valence of social information (either positive or negative compared to an absence of social information) modulates early neural components associated with visual processing and linked with activity of visual and face areas (P100 and N170, respectively). Given that the impact of social information on later components such as LPP was already and extensively shown elsewhere (Schupp et al., 2006; Bublatzky et al., 2014), we focused our analysis on the early ERP components in order to provide evidence that social information modulates not only high-level cognitive functions such as attention and executive functions, but also *perception and categorization* of emotional stimuli.

The P100 component, occurring ~120 ms after the stimulus onset, is associated with the processing of exogenous visual stimulation in extrastriate cortical areas (Clark and Hillyard, 1996), suggesting that the P100 is modulated by the physical properties of the stimulus. With regards to EFE processing, the exogenous nature of the P100 component has been supported by previous studies showing that fearful emotions generated larger amplitudes as compared to neutral emotions (Pourtois et al., 2004, 2005). However, the emotional stimuli used in these studies were different at the emotional (endogenous) and at the perceptual (exogenous) level. For instance, angry vs. happy faces differ at the emotional level, but they are also considerably different at a purely perceptual level, even after a careful control of luminance or contrast information. Therefore, the modulation of the P100, as well as the modulation of N170 component (related to facial processing) by endogenous variables, (e.g., social information) remains a matter of debate. Thus, in addition, the current study also aimed to determine whether the P100 and N170 components could be modulated by endogenous social factors applied on *strictly identical EFE at a perceptual level*. Furthermore, at a physiological level, we aimed to evaluate whether mimicry processes, assessed by EMG responses to EFE, are modulated by social information.

## Hypotheses

**Study 1** examined the effect of a positive, negative, or no social information on participants' ratings of the valence and intensity of EFE. We expected significantly higher ratings of valence and intensity when the valence of the EFE was congruent with the valence of the social label (e.g., joy expressed by nice nurse) compared to the control situation without social information. Conversely, we expected lower ratings of valence and intensity for a given EFE when the valence of the EFE and the social label (i.e., joy expressed by a serial rapist) was incongruent compared to the control situation without social label. We also examined how the emotion *felt* by the participants was modulated by social information. Indeed, it has been shown that the social information (i.e., ethnicity) about the target depicted in the EFE modulates empathic emotional responses (Drwecki et al., 2011).

**Study 2** investigated the impact of social information on EMG activity. We recorded EMG over six facial muscles involved in specific expressions, such as the contraction of the zygomaticus major (i.e., in response to happiness) and the corrugator supercilii (i.e., in response to anger). We expected increased EMG activity of the corresponding muscle when the facial expression was preceded by social information congruent to the emotion displayed by the stimulus compared to no social label. For example, we expected that congruency trials (i.e., happy facial expression with a positive label) should elicit higher EMG activity in muscles involved with producing facial expressions of joy (zygomaticus major and orbicularis oculi) compared to the same EFE presented without social information. This hypothesis is based on previous research showing that social factors may increase facial mimicry of likable targets or in-group members (Mondillon et al., 2007; Likowski et al., 2008). Conversely, incongruent trials (e.g., happy facial expression with a negative label) should produce lower EMG activity of the corresponding facial muscles (zygomaticus major and orbicularis oculi) compared to the same EFE without social information.

**Study 3** examined the potential effects of social information on two early visual ERP components, namely the P100 and N170. Based on the findings of Achaibou et al. (2008), showing early effects of dynamic emotional expressions on mimicry and neural activity assessed by P100 and N170 components, we expected higher amplitudes of the P100 and N170 when the EFE is congruent with the preceding social information compared to the control situation in which no social information is provided. Our hypothesis is based on a similar assumption of top-down higher-level processes influencing early neural and facial responses. However, the current study manipulates social information rather than emotional expressions, thus ensuring that bottom-up perceptual factors are strictly identical.

## Study 1

The influence of social context on the processing of emotional faces was first investigated at the subjective level. The goal was to examine the effects of social context on the ratings of valence and intensity of the EFE as well as the emotions felt in response to these EFE.

### Method

#### Participants

Twenty-four undergraduate students (19 females) (*M*_*age*_ = 20.50, *SD*_*age*_ = 2.54) at the University Clermont Auvergne, Clermont-Ferrand (France) with corrected-to-normal vision, participated in exchange for course credits. All participants gave written informed consent and had normal or corrected vision and no psychiatric or neurological disorders.

#### Material and procedure

##### Social information

The social context of EFE was operationalized with verbal labels referring to positive and negative categories. Each label was composed of two words (noun and adjective; e.g., loving mother). Thirty-two labels were initially created: 16 positive and 16 negative adjectives referring to either male or female nouns (see Appendix). A pilot study was carried out with 24 undergraduate psychology students (20 females; *M*_*age*_ 20.70; *SD*_*age*_ = 2.54) to test the pleasantness and unpleasantness of all labels. For each label, participants rated the extent to which the person described by the label was pleasant or unpleasant with two 7-point Likert scales for pleasantness and unpleasantness (ranging from 0 to 6; not at all to totally). The six labels rated as most pleasant (i.e., loving mother, passionate teacher, humanitarian doctor, nice nurse, cheerful sportsman, caring father; *pleasant scale*: *M* = 5.76, *SD* = 0.29; *unpleasant scale: M* = 2.06, *SD* = 0.37) and six rated as most unpleasant (i.e., dangerous schizophrenic, sadistic killer, serial rapist, asocial necrophiliac, brutal coach, pedophile therapist; *pleasant scale*: *M* = 1.63, *SD* = 0.49*; unpleasant scale: M* = 6.30, *SD* = 0.58) were retained for the experiments. Subsequent analysis showed that positive labels significantly differed from negative labels in terms of pleasantness [*t*_(10)_ = 17.69, *p* < 0.001] and unpleasantness [*t*_(10)_ = −15.12, *p* < 0.001].

##### Dynamic EFE

**Figure 1:**
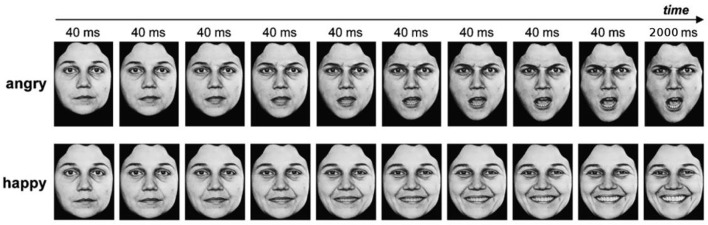
Example of dynamic facial expressions. Copyright available in Ekman and Friesen (1976). *Pictures of facial affect*. Palo Alto, CA: Consulting Psychologists Press.

The dynamic faces expressed a neutral emotion and then either gradually changed to express the full intensity of six basic emotions (anger, disgust, fearful, happiness, sadness, and surprise; Ekman and Friesen, 1976) or remained neutral (no emotion). For each emotional category, there were 10 different face identities (4 females and 6 males). We aimed to balance as much as possible the gender of the stimuli (given the database available) since the gender of the emotional expression, irrespective of the gender of the participant, might interact with the expressed emotions: notably, female faces tend to be perceived as happier and male faces as angrier (Villepoux et al., 2015). This made a total of 70 different movie clips (10 identities x 7 EFE). For a reliable control of the onset, duration, and intensity of emotional expressions, we used dynamic expressions from a set of morphed pictures from a neutral to an emotional expression of the same face identity on the basis of Benson and Perrett's morphing technique (Benson and Perrett, 1993). Thus, we used 18 frames per face with increasing emotional intensity for each emotion. Each of the first 17 frames was exposed for 40 ms and the 18th frame lasted for 2,000 ms (Figure 1).

#### Procedure

Participants were seated in front of a computer screen on which the dynamic EFE was displayed in central vision. EFE pictures were presented using the E-Prime software (Psychology Software Tools, http://www.pstnet.com) creating the compelling illusion of a short movie clip displaying a dynamic facial expression from neutral to emotional expressions. The experiment was divided into two phases. In the first experimental phase, participants were exposed to a central fixation cross for a duration varying from 500 to 1,000 ms, followed by a video presenting one EFE (2,680 ms). After each of the 70 videos, participants were instructed to rate the EFE in terms of valence on a continuous pixel scale ranging from 0 (positive) to 100 (negative). Next, participants rated the intensity of the EFE on each of the six basic emotions (anger, disgust, fear, happiness, sad, or surprise) on an identical pixel scale (ranging from 0: not at all, to 100: totally). The second phase was similar to the first phase, except that each of the 70 videos was preceded by a positive or negative label. A specific identity from the database (for every EFE expressed by this individual) was associated with a positive label (e.g., nice nurse) for half of the participants, and a negative label for the other half (e.g., serial rapist). The social valence of each identity was counterbalanced across participants. After seeing the label displayed on the screen for 2,000 ms, participants were exposed to a fixation cross (from 500 to 1,000 ms) before each video depicting a specific EFE of a specific person (for 2,680 ms). Similarly to phase 1, participants had to assess the valence and the intensity of the *perceived* EFE. Moreover, participants were also instructed to evaluate the emotion they *felt* after exposure to each video on the same continuous scales as Phase 1 (i.e., valence and intensity). The presentation order of movie clips was randomized for each phase.

### Statistical analyses

All the statistical analyses were performed using the STATISTICA 7 software. We conducted an analysis of variance (ANOVA) on valence and intensity of the emotion *perceived* (Phase 1 and 2) and valence and intensity of the emotion *felt* by the participant (for Phase 2 only) as the dependent variables, and EFE (Anger; Disgust; Fear; Happiness; Sad; Surprise or Neutral) and social label (Phase 1—no social label; Phase 2—negative social label; Phase 2—positive social label) as within-subjects independent variables. Concerning the emotional intensity of EFE, for simplicity and clarity reasons, we only kept the intensity level related to each emotion (e.g., anger responses after presentation of an angry faces) and not the entire confusion matrices.

### Perceived valence

At the level of valence, we observed a significant two-way interaction, *F*_(10, 230)_ = 5.38, *p* < 0.001, between social label and EFE (Table 1). Given the number of potential combinations of pairwise comparisons, we did not conduct *post-hoc* analyses. However, descriptive statistics provided in Table 1 illustrate this interaction by showing that positive emotions were perceived as less positive after exposure to negative compared to the situation without social label. Conversely, negative emotions were perceived as less negative for actors presented with a positive compared to an absence of social information.

**Table 1:** Average rated level of perceived valence, from 0 (negative) to 100 (positive), of each emotional expression depending on the valence of the label (negative, positive, or no label).

**Displayed EFE**	**Social label**		
	**No social valence**	**Negative social valence**	**Positive social valence**
Anger	9.19 (1.87)	10.96 (2.18)	16.77 (2.42)
Disgust	11.08 (2.08)	12.15 (2.05)	20.05 (2.34)
Fear	12.26 (2.07)	14.90 (2.53)	18.55 (2.93)
Happy	92.46 (1.22)	81.40 (3.92)	90.24 (1.99)
Sad	11.92 (1.71)	17.80 (2.46)	18.26 (2.37)
Surprise	43.16 (2.71)	40.63 (2.97)	45.59 (2.66)

*Standard errors are indicated into brackets*.

### Perceived emotional intensity

Analyses of the intensity of the perceived emotional expressions revealed a significant two-way interaction, *F*_(10, 230)_ = 2.01, *p* < 0.05, between social label and EFE (Table 2) on the intensity of the corresponding emotion. Again, pairwise comparisons were not significant after *post-hoc* corrections but Table 2 illustrates this interaction and reveals that positive emotions were perceived as less intense when accompanied by negative compared to the situation without social label. Conversely, negative emotions were perceived as less intense for actors presented with a positive compared to an absence of social information.

**Table 2:** Average ratings of intensity of *perceived* emotional expression, from 0 (not expressed) to 100 (fully expressed), as a function of the valence of labels (negative, positive, or no labels).

**Displayed EFE**	**Social label**		
	**No social label**	**Negative social label**	**Positive social label**
Anger	69.43 (3.7)	70.00 (3.37)	63.59 (3.88)
Disgust	57.27 (3.4)	45.22 (5.5)	43.09 (4.07)
Fear	59.10 (3.68)	54.70 (4.08)	50.29 (4.97)
Happy	87.22 (1.69)	81.47 (3.39)	86.40 (2.51)
Sad	60.25 (4.05)	59.35 (4.71)	56.85 (4.87)
Surprise	83.40 (2.88)	76.24 (3.95)	77.59 (3.97)

*Standard errors are indicated into brackets*.

### Felt valence

We observed that the social valence of the stimulus affected the participants' responses concerning felt valence. More precisely, results point to a significant interaction between the social information and EFE [*F*_(5, 115)_ = 20.6, *p* < 0.001]. As for perceived valence, negative labels were associated with more negative feelings than were positive labels for negative emotions. Conversely, positive labels were associated with more positive feelings than were negative labels for positive emotions (Table 3).

**Table 3:** Average rated level of felt valence, from 0 (negative) to 100 (positive), of each emotional expression depending on the valence of the label (negative vs. positive).

**Displayed EFE**	**Social label**	
	**Negative social label**	**Positive social label**
Anger	18.38 (3.14)	32.49 (3.31)
Disgust	17.82 (2.34)	35.48 (3.28)
Fear	28.07 (3.71)	33.87 (2.49)
Happy	44.75 (4.48)	81.04 (3.09)
Sad	29.54 (3.26)	34.50 (2.81)
Surprise	37.68 (2.95)	50.55 (3.02)

*Standard errors are indicated into brackets*.

### Felt emotional intensity

We also found a significant interaction between social label and EFE [*F*_(5, 115)_ = 17.80, *p* < 0.001] regarding the intensity of corresponding emotion felt by the participants. For instance, participants perceived negative emotions as more intense after negative than after positive labels. Conversely, participants reported a higher level of positive emotions after positive labels than after negative labels (Table 4).

**Table 4:** Average ratings of intensity of *felt* emotion, from 0 (not expressed) to 100 (fully expressed), as a function of the valence of labels (negative vs. positive labels).

**Displayed EFE**	**Social label**	
	**Negative social label**	**Positive social label**
Anger	22.77 (5.44)	11.95 (4.74)
Disgust	31.74 (5.72)	17.17 (4.81)
Fear	20.38 (5.32)	20.01 (5.88)
Happy	20.09 (5.82)	53.73 (6.31)
Sad	19.10 (4.90)	31.72 (4.79)
Surprise	18.83 (5.37)	21.42 (5.80)

*Standard errors are indicated into brackets*.

### Discussion

With regards to *perceived valence* of facial expressions, this study showed that expressions of negative emotions were perceived as less negative when preceded by a positive label compared to a neutral situation whereas positive emotions were rated as less positive when preceded by a negative label compared to an absence of social information. We observed a similar interaction effect modulating the ratings of the *intensity perceived* of the emotional expressions, negative emotions were perceived as less intense when preceded by positive social labels, and conversely for a negative social information. Moreover, we observed the same interaction effect for the *valence of the emotion felt* but also in the ratings of the *felt emotional intensity*.

This study thus supports hypothesis 1 according to which target-related social information influences the ratings of valence and intensity of the EFE as well as the emotions felt in response to these EFE. More precisely, incongruent social terms reduce the evaluation of valence and intensity of the EFE and congruent trials led to higher intensity of felt emotions. Moreover, incongruent trials led to a lower valence of felt emotions compared to a control situation of perceiving emotional expressions without social information. We thus extend upon previous findings suggesting that top down social factors influence EFE processing (e.g., Kubota and Ito, 2007; Likowski et al., 2008; Bublatzky et al., 2014). In terms of affective responses to EFE, we support Drwecki et al.'s (2011) findings that emotional responses are influenced by social information (i.e., ethnicity) about the target depicted in the EFE.

More globally, this study is in line with previous articles showing the influence of participants' appraisal of the target on the emotional evaluation and responses to EFE. For instance, Lamm et al. (2007) assessed how reappraisal might influence participants' evaluation of the pain expressed by faces of patients undergoing painful treatment. When participants were told that the treatment was effective (vs. non-effective), they judged the experience of the person as being less painful. Results also showed that participants from the “non-effective” group reported higher distress than those from “effective” group. Therefore, in line with Lamm et al.'s (2007) results, the present findings showed for the first time that appraising EFE as socially congruent or incongruent with the expression modulates their evaluation as well as their emotional effects.

Study 2 and 3 aim to determine the neural and physiological processes underpinning the modulation of social information of EFE on behavioral responses. According to the embodiment theory, we assume that the embodied processing of EFE might be modulated by social information, such that participants might have simulated to a lesser extent the EFE associated with incongruent social information.

## Study 2

In this experiment, we investigated the impact of social information on mimicry within the theoretical framework of the embodiment theory. More specifically, we expected increased EMG activity in the corresponding muscles when EFE was preceded by a congruent social label compared to an EFE without social label. Conversely, incongruent trials should produce lower EMG activity of the corresponding facial muscles compared to the same EFE with no social label.

### Method

#### Participants

Twenty healthy subjects (19 females) (*M*_*age*_ = 20.80 years, *SD*_*age*_ = 2.26) at the University Clermont Auvergne, Clermont-Ferrand (France), with corrected-to-normal vision, participated in exchange for course credits. All participants gave written informed consent and had no psychiatric or neurological disorders.

#### Material and procedure

The experimental design of Study 2 is similar to Study 1, except that we recorded participants' EMG activity during the experimental task. Electrodes were fixed on the participant's face in order to record facial muscle electrical activity (EMG). Before attaching the electrodes, the skin was cleaned with alcohol in order remove sebum. Six pairs of electrodes were placed on the following muscle regions (see Fridlund and Cacioppo, 1986 for details): frontalis pars medialis (mainly related to surprise and fear), corrugator supercilii (mainly related to anger), orbicularis occuli pars orbitalis (mainly related to happiness), levator labii superioris (mainly related to disgust), zygomaticus major (mainly related to smile), and orbicularis oris inferior (mainly related to sadness). The reference electrode was clipped on the ear lobe. Because of the large electrode size, both sides of the face were required. Half of the electrodes (corrugator, levator, and orbicularis oris) were placed on the right side of the face while the other three electrodes (frontalis, orbicularis occuli, zygomaticus major) were placed on the left side. The placement of the electrodes was identical for all participants. Locations for each specific muscle were chosen to allow the setting of the six pairs of electrodes while (i) allowing the minimum of overlap between close muscles (e.g., corrugator and levator) and (ii) use the most responsive right side of the face (Fridlund and Cacioppo, 1986) for the weakest muscles (corrugator, levator, and orbicularis oris). After checking the position of each pair, study instructions were given to the participants and the experiment began.

#### Data acquisition

Each EMG signal was acquired by bipolar electrodes used for electrophysiological acquisition and amplified by Multi-Channel Bio Amps GT201 band pass filtered from 10 to 1,000 Hz (ADInstruments equipment, ML880 Powerlab 16/30). We used the absolute value of the EMG signal. The EMG data were averaged during the 5,000 ms after stimulus onset. The dependent variable of interest was measured as the difference between the mean activity 5,000 ms after the stimulus and the baseline activity recorded during the 500 ms before the stimulus onset, when a fixation cross was displayed on the screen.

#### Statistical analyses

All analyses were performed using PASW Statistics 18 (SPSS Inc., Chicago, IL). We conducted an analysis of variance (ANOVA) with EMG activity (6 levels: frontalis pars medialis, corrugator supercilii, orbicularis occuli pars orbitalis, levator labii superioris, zygomaticus major, and orbicularis oris inferior) as dependent variables and EFE displayed on screen (Anger; Disgust; Fear; Happiness; Sad; Surprise or Neutral) and social label (no social label; negative social label; positive social label) as within-subjects independent variables. Corrections of Greenhouse-Geisser were also applied for variance analysis when violations of sphericity occurred.

### Results

**Figure 2:**
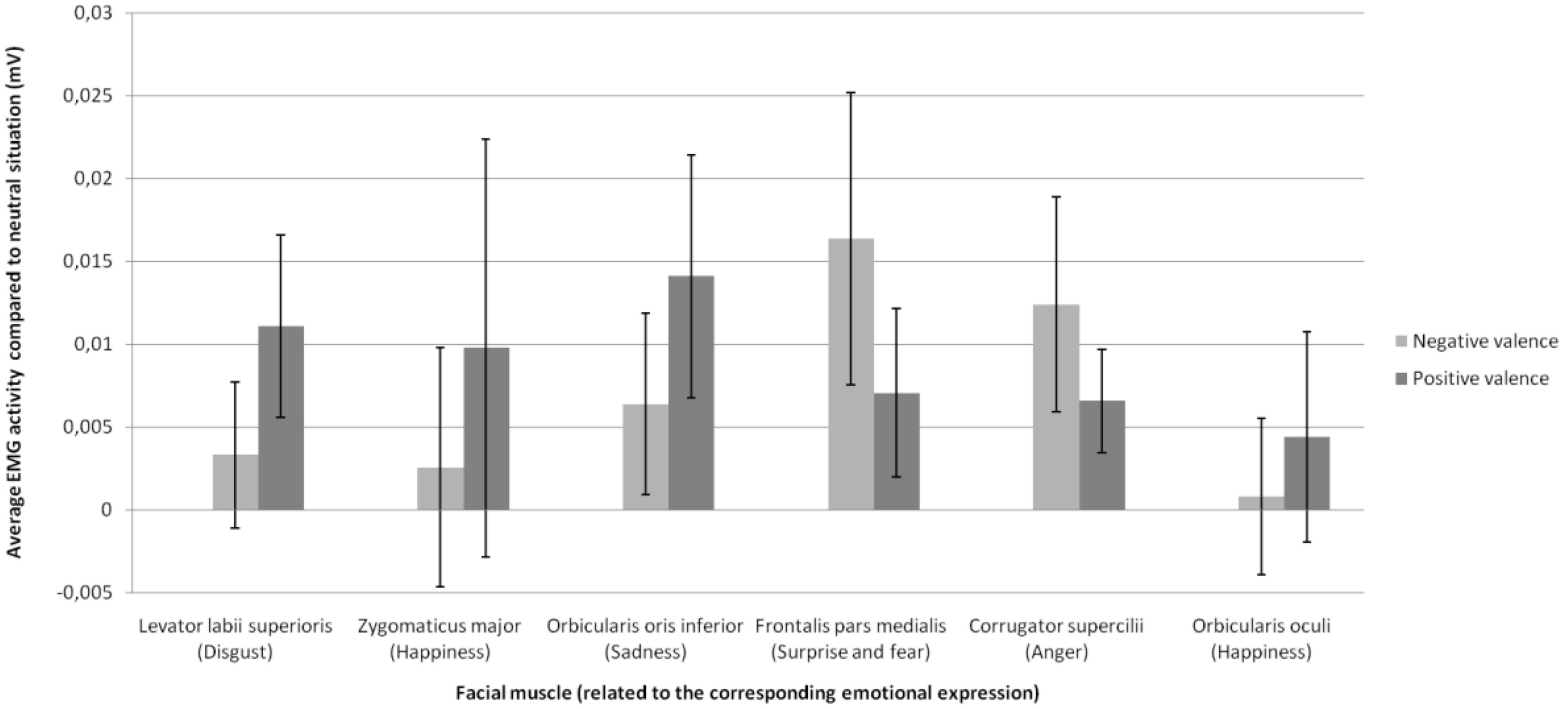
Mean change in EMG activity (compared to neutral situation without social information) as a function of emotional expression on the facial muscle related to this emotional expression for each social valence of the stimulus.

We observed a significant interaction between emotion and muscle, *F*_(5.54, 105.22)_ = 2.37, *p* < 0.05, confirming that each emotion was related to a specific set of muscles (e.g., zygomaticus major with joy, corrugator supercilii with anger). Therefore, we focused the analyses on the muscles specific to each emotion (Figure 2). Statistical analyses revealed a significant main effect of social label, *F*_(1.36, 25.36)_ = 8.32, *p* < 0.01. As shown on Figure 2, labels of positive and negative social valence produced a significant increase (relative to baseline) in EMG activity in response to EFE compared to the EMG activity elicited by EFE displayed to the control situation without social information. Negative [*t*_(19)_ = 3.34, *p* < 0.01, two-tailed *t*-test] and positive [*t*_(19)_ = 2.83, *p* < 0.05, two-tailed *t*-test] social labels produced a significant increase in EMG activity compared to control situation without social labels, while the effect of positive and negative social labels on EMG activity did not differ [*t*_(19)_ = 0.80, *p* = 0.93, two-tailed *t*-test]. No main effect was observed for either emotion or muscle, and interaction effects (emotion x social label; muscle x social label; muscle x emotion x social label) were not significant. Therefore specific pairwise comparisons were not analyzed.

### Discussion

Whereas we hypothesized that social information will modulate facial mimicry in response to congruent EFE, we found a significant *increase of EMG activity* when EFE were preceded *by both positive and negative social label* compared to the control situation without social information. Contrary to the behavioral response reported in Experiment 1, the influence of social information on EMG responses was thus *independent* of congruency between the social label and the EFE. Therefore, these findings suggest that the mimicry process is significantly enhanced by the mere presence of social information, independently of its valence.

In respect to the main effect of social information, we supported the hypothesis that social information constitutes a top-down factor that can modulate physiological responses to emotional expressions. This study is thus in line with previous findings that mimicry of smiles is enhanced in the presence of friends but not in the presence of strangers (Hess et al., 1995) and in the presence of in-group members (Mondillon et al., 2007; Niedenthal et al., 2010; Wang and Hamilton, 2012). In this study, we showed for the first time that social information about the expresser of a given EFE also modulates their processing.

However, we did not show an interaction between social information and EMG responses to EFE, thus failing to extend the behavioral findings of Study 1 to EMG activity and to support the modulatory impact of (in)congruency. Several reasons might explain the absence of interaction between (in)congruency condition and EFE. First, while some studies found an association between subjective and expressive responses to emotional stimuli (Schwartz et al., 1980) others failed to find such effect (e.g., Sloan et al., 1997; Calder et al., 2000; Rottenberg et al., 2002). For instance, although spontaneous mimicry leads to greater affective empathy while watching people expressing facial emotions (Stel and Vonk, 2010), Calder et al. (2000) showed that, despite their facial paralysis, patients with a Möbius syndrome accurately recognize facial expressions. Therefore, EMG activity induced by social labels may not be the main determinant of subjective response found in Study 1. Second, the present findings may suggest that significant increase in EMG activity constitutes a physiological mechanism that allows an enhanced processing of the relevant social information, beyond the object of this information. In other words, the social relevance of individuals may be processed prior to their emotional state. This may elicit bodily reactions to social context, no matter the emotional state of the targets.

## Study 3

Social information modulated the subjective (Study 1) and physiological responses (Study 2) to emotional expressions in a different manner (irrespective of congruency in Study 2). Study 3 aimed to examine whether early neural responses to EFE (i.e., P100 and N170 components) are modulated by social context. We hypothesized that the subjective and physiological modulations observed in Studies 1 and 2, respectively, could be related to a modulation of rapid neural components related to visual perception. More precisely, we expected that social information might modulate P100 and N170 components in response to EFE in a way congruent with the social information provided to the participant. Our hypothesis also extends the results reported by Achaibou et al. (2008) indicating that facial mimicry (assessed by EMG activity) is related to P100 and N170 EEG components. Precisely, we expected higher amplitudes of the P100 and N170 in response to congruent trials (e.g., happy face following positive social information) compared to the control trials (no social label). Conversely, we expected lower amplitudes of the P100 and N170 in response to incongruent trials (e.g., happy face following negative social information) compared to the control trials (no social label).

### Method

#### Participants

Fifteen healthy subjects (10 females) (*M*_*age*_ = 21.67; *SD*_*age*_ = 6.64) from University Clermont Auvergne participated in this experiment in exchange for course credits. All participants gave written informed consent and had normal or corrected vision and no psychiatric or neurological disorders.

#### Stimuli and procedure

The stimuli were identical to those used in Study 1 and 2. The procedure was also similar except (a) that EEG were recorded during the experiment, (b) that the number of presented blocks was increased, and (c) that emotional intensity and valence of the EFE were only rated after the first block for phase 1 (composed of 3 blocks in total) and after the first block for phase 2 (composed of 4 blocks in total). The subsequent blocks for each phase required only passive observation of the EFE (i.e., without subjective responses). Thus, seven blocks were presented to the subjects, including 3 blocks for phase 1 with EFE without social labels (control situations) and 4 blocks for phase 2 with EFE preceded by a social label (either positive or negative). The participants were placed in front of the same screen (CRT 17”) as in Study 1, but were fitted with the EEG electrodes. EEG recordings were performed during the five blocks of passive exposure to EFE stimuli.

#### Data acquisition

Scalp EEG was amplified using the BIOSEMI Active-Two amplifier and was recorded from 64 electrodes distributed on an elastic cap. The distribution of electrodes was made according to the EEG 10–20 system: the electrodes included Fp1, AF3, AF7, F1, F3, F5, F7, FC1, FC3, FC5, FT7, C1, C3, C5, T7, CP1, CP3, CP5, TP7, P1, P3, P5, P7, P9, PO3, PO7, O1 for the left hemisphere; the equivalent electrodes for the right hemisphere; and FPz, AFz, Fz, FCz, Cz, CPz, Pz, POz, Oz, Iz for the electrodes of the conventional midline sites. Two other electrodes, CMS (Common Mode Sense) and DRL (Driven Right Leg) were respectively used as electrodes of reference and mass. CMS was the active electrode while DRL was the passive electrode. Data were filtered with a low-pass filter of 200 Hz and digitized with a sampling rate of 512 Hz.

**Figure 3:**
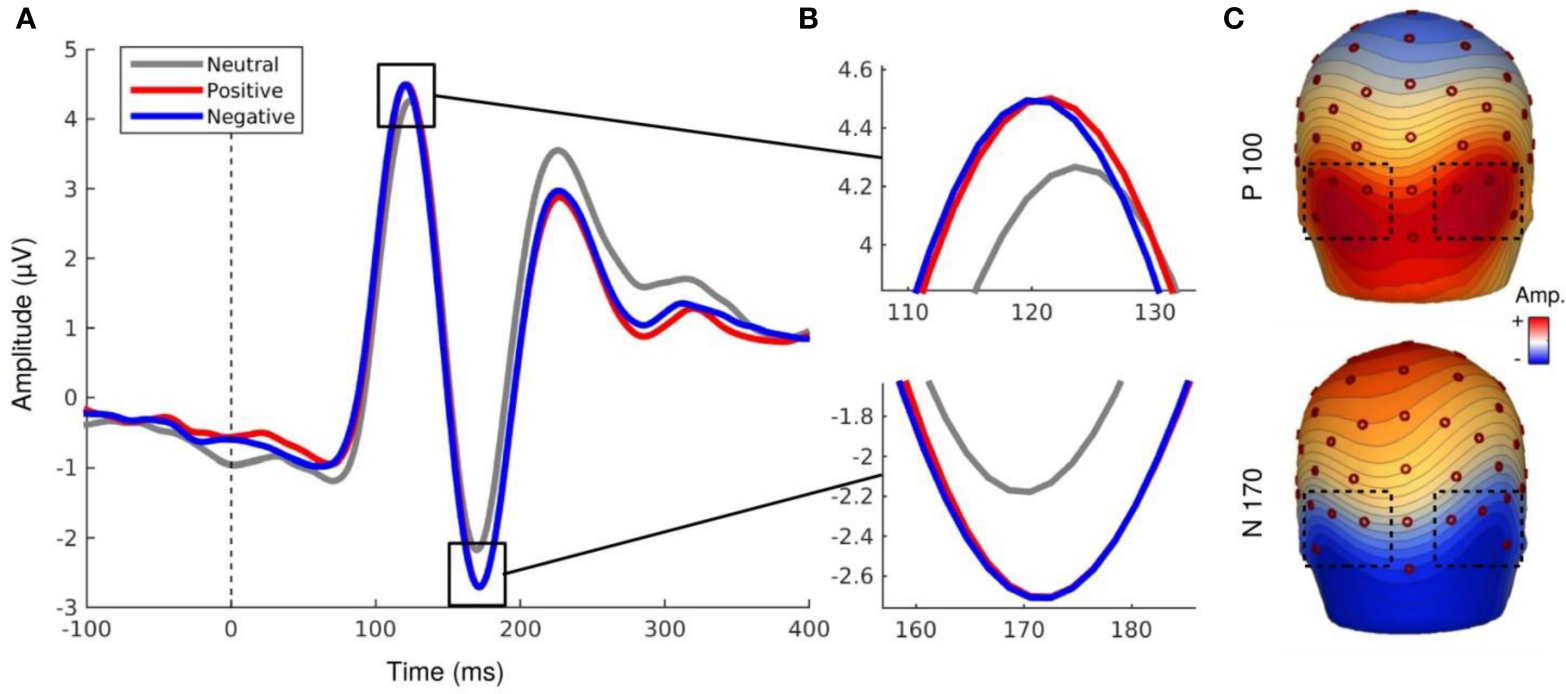
**(A,B)** EEG activity for the P100 and N170 components. **(C)** Topography map of P100 (121.90 ms, 0.5 μV by color gradients) and N170 component (167 ms, 0.31 μV by color gradients).

EEG signal processing was performed with the software BESA (http://www.besa.de/). The data were all re-referenced to the average reference then filtered with a 1–40 Hz pass-band filter. For every recording of each subject, a correction of artifacts (such as eye blinks) was applied. BESA allows to manually identify artifacts for every subject and then automatically generalizes this removal of artifacts for the entire recording. Trials were epoched from −200 to +1,000 ms with respect to the stimulus onset. After averaging, we obtained the peaks of the components using BESA. ERPs were averaged for every subject and every condition, and corrected using a baseline of 200 ms prior to the stimulus onset. Finally, grand averages were computed across subjects for all the conditions. The P100 and N170 components were studied on the cluster of electrodes where their amplitudes were the highest: O1, O2, PO7, PO8, P7, P8, P9, P10. Mean amplitudes of P100 (time window: 100–140 ms) and N170 (time window: 150–190 ms) were analyzed for each condition. Peak latency extracted with BESA was at the mean latency of 121.90 ms for the P100 component and the mean latency of 167 ms for the N170 component. The topographic map of each component was provided on Figure 3.

#### Statistical analyses

All the statistical analyses were performed using the STATISTICA 7 software. Mean amplitudes of P100 were analyzed using repeated-measures ANOVA. Mean amplitudes of both components were analyzed as a function of displayed Emotion (7 levels: neutral, anger, sadness, enjoyment, disgust, surprise, and fear), Social Label (3 levels: no social label, positive social label, negative social label) and Hemisphere (2 levels: right, left). Greenhouse-Geisser corrections were applied for variance analysis when violations of sphericity occurred (ε referred to as Greenhouse-Geisser estimate epsilon). The condition of sphericity was verified by a test of Mauchley.

### Results

#### P100 component

Analyses revealed a main effect of social label, *F*_(1.43, 20.02)_ = 5.76, *p* < 0.02, ε = 0.71, indicating that the mean amplitudes of the P100 component differ as a function of the associated social label (Figure 3). EFE preceded by social label, regardless of its valence, elicited larger amplitudes of the P100 compared to EFE presented without social label (but did not differ between positive and negative label). Neither the hemisphere, nor emotion effects were significant. However, the interaction Hemisphere x Emotion was significant, *F*_(6, 84)_ = 2.49, *p* < 0.03, indicating higher amplitudes of P100 on the right hemisphere for specific emotions (but *post-hoc* corrected pairwise comparisons were not significant). No other interaction was significant.

#### N170 component

**Figure 4:**
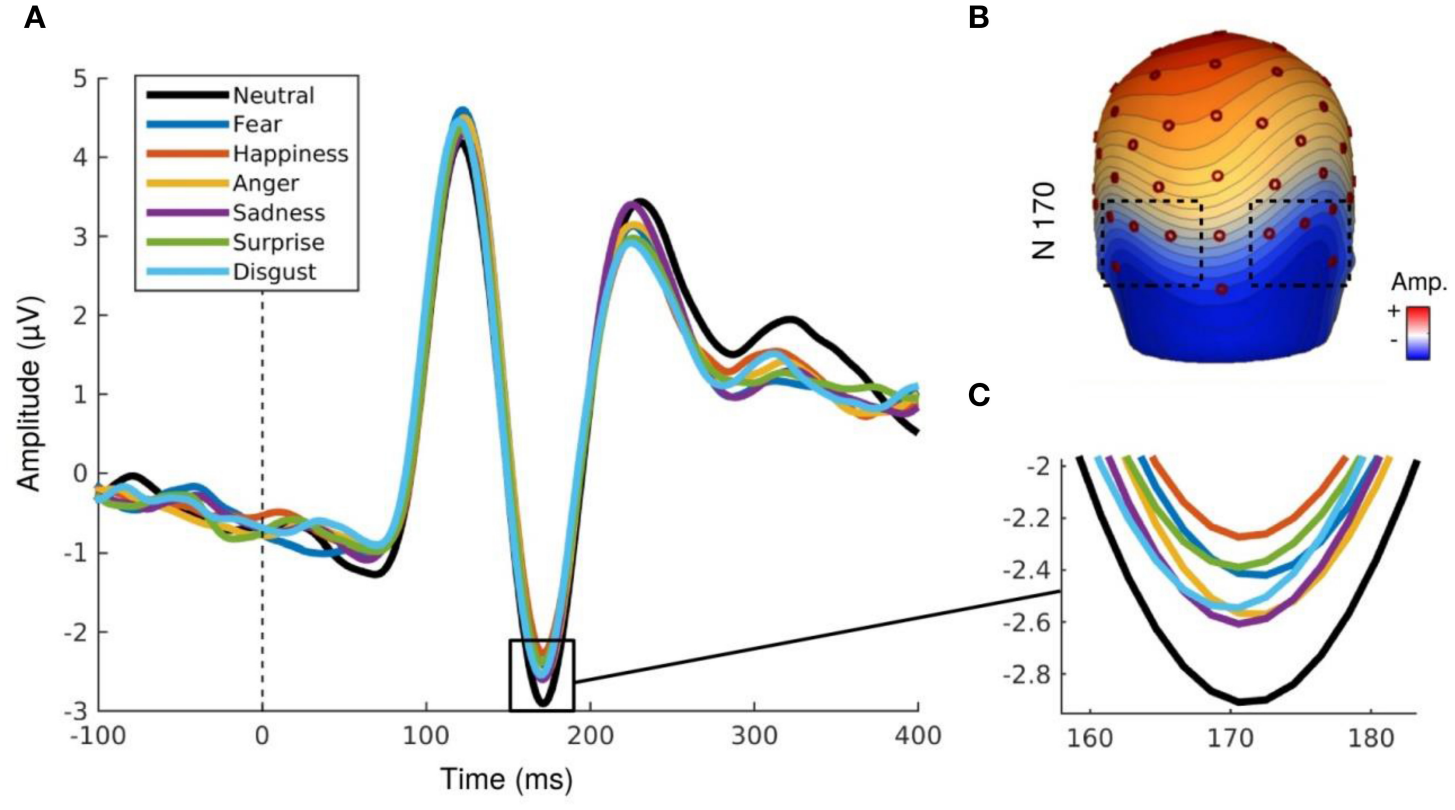
**(A,B)** N170 component for each emotional expression irrespective to social valence. **(C)** Topography map of N170 component (167 ms, 0.31 μV by color gradients).

Similarly to the P100 component, analyses of the N170 component revealed a main effect for social label, *F*_(1.29, 18.13)_ = 5.47, *p* < 0.03, ε = 0.64. The amplitude was significantly higher for EFE when preceded by positive and negative social label than for EFE without social label (Figure 3) but there was no significant differences between positive and negative social labels. We also observed a main effect for emotion, *F*_(6, 84)_ = 4.15, *p* < 0.002, indicating that the N170 amplitudes differed significantly as a function of displayed facial expression (Figure 4). Analyses showed neither a main effect of the hemisphere, nor any other interaction effect.

### Discussion

Our results indicate a modulation of the N170 component depending on emotion. More importantly, we observed higher amplitudes of the P100 and N170 in response to trials associated with a social information compared to control trials (without social label). However, contrary to our initial hypothesis, there was no significant interaction between social label and emotional expressions. Rather, electrophysiological data indicate a main effect of social label on both P100 and N170 components compared to the control situation and irrespective to the valence (and therefore, congruency) of the social label associated with any of the EFE. This effect was observed for the N170, and largely associated with cognitive processes involved in recognition of categorization of human faces (Bentin et al., 1996). Even more surprising, we observed a modulation of the P100 component which is associated with basic visual perceptual processing (Allison et al., 1999) confirming that this basic perceptual component could be modulated by endogenous variables (Pourtois et al., 2005). In other words, we confirm and extend previous findings that point to a modulation of rapid neural components to higher-level (but perceptually identical) endogenous variables related to social information.

Moreover, this modulation quantitatively increased the neural processes associated with perceptual processing of EFE of the specific individuals but did not qualitatively modulate these processes in regards to the congruency between the social valence and the emotion displayed by the face. Thus, this study is in accordance with the findings of Study 2, which showed a significant increase of EMG activity when EFE were preceded by both positive and negative social label, *irrespective* of the congruency between the social label and the EFE.

## General discussion

### Influence of social context on subjective, physiological, and neural activities

The aim of this article was to investigate whether social information about the target expressing a facial emotion could modulate the processing of EFE at subjective, physiological, and neural levels. Specifically, we aimed to evaluate the effect of valence congruency between social information about targets and their EFE on the processing of these EFE. The study was based on previous findings showing that perceptual-social characteristics of the expresser (e.g., ethnicity) influence the processing of black vs. white emotional expressions for N100 to P300 components (e.g., Kubota and Ito, 2007). However, no study up to date had investigated the effect of higher-level social information about the target on EFE processing by maintaining *strictly identical the perceptual features*.

In Study 1, we supported our hypothesis that social information modulates EFE processing at a subjective level. Indeed, we showed that incongruent valence between social information and EFE reduces the valence and intensity of perceived and felt emotions. Specifically, we showed that negative expressions were perceived as less negative and less intense when preceded by a positive label compared to no label. Conversely, a positive facial expression was rated as less positive and less intense when preceded by a negative label compared to no label. Our results thus extend previous findings on modulation of EFE recognition by social factors (e.g., Kubota and Ito, 2007; Mondillon et al., 2007). We show that higher-level social information may impact EFE processing. This finding supports the influence of top-down information (extended to higher-order social information and crucially, strictly identical perceptually) on emotional processing of EFE.

As concerns the results of Study 2 examining EMG responses to facial expressions, our results build on and extend recent models of embodied cognition assuming that circuits of mimicry are preferentially activated when the observed emotion is socially relevant (Niedenthal et al., 2010; Wang and Hamilton, 2012). More specifically, previous data pointed to a significant modulation of embodiment circuitry by social information such as the presence of friends, but not strangers (Hess et al., 1995) or in the presence of in-group compared to out-group members (Mondillon et al., 2007). In the present study, we found weak general EMG activity (including the muscle associated with each specific EFE) when facial expressions were presented without any social label (control condition). However, contrary to our hypotheses, we found a significant increase of EMG activity when expressions were preceded by both positive and negative social label, irrespective of the congruency between the social label and the EFE. This suggests that embodiment circuitries may act as a boost for subsequent cognitive or emotional processes when the situation is socially relevant for the individual, but irrespective of its specific content (either negative or positive social situations). This supports and extends previous articles suggesting a predominant role of arousal (compared to valence) during embodiment of emotional cues (Beffara et al., 2012, 2016; Kever et al., 2015).

In Study 3, our goal was to determine whether this non-specific boost observed at a psychophysiological level could be related to a modulation, at a neural level, of low level perceptual processes. Our results indicate a main effect of social information as positive and negative social information produced higher amplitudes of P100 and N170 components compared to situations without social information. Therefore, Study 3 extends previous results reported by Achaibou et al. (2008) pointing to a modulation of the P100 and N170 by EFE in relation to EMG activity and demonstrates their relevance to higher-level social information. Our results are also in line with other findings showing that top-down factors related to the target may modulate neural responses to EFE (Kubota and Ito, 2007; Tortosa et al., 2013). Finally, they also support fMRI studies that have shown that social information modulates the neural correlates of facial expression (e.g., Winston et al., 2002; Singer et al., 2004; Vrtička et al., 2009; Cloutier et al., 2012). However, similarly to Study 2 and contrary to our initial hypotheses, this effect was independent on the congruency between social information and EFE.

## Limitations and perspectives

Our findings extend the current state-of-the-art by revealing that the observed top-down influences cannot be explained by bottom-up perceptual factors (e.g., the perceptual difference between happy and angry faces) since our stimuli were strictly identical at a perceptual level (e.g., an identical smile expressed by a negative vs. positive individual). Moreover, our findings revealed that social information can influence low level perceptual processes of EFE. Therefore, these findings demonstrate that social context has an influence, as early as 120 or 170 ms after onset, on low-level perceptual and cognitive processing of emotional expressions.

However, although Study 2 and 3 support a top-down influence during visual perception of emotional stimuli, it remains to be tested whether increased physiological and neural reactions after social information priming are specific to social information or could be accounted for by either conceptual or other social factors. Future studies should thus include other control situations in order to understand whether the effects reported in the current study are specific to social information contrasted in valence. We can imagine that other types of social (e.g., group membership, discriminated individuals) but also more general conceptual information could produce similar patterns of rapid physiological and neural modulation. Finally, one could argue that EMG activity also results from higher relevance for the self (i.e., involving the observer) during social vs. no social priming (Grèzes et al., 2013).

Moreover, although we did not examine the neural activity with a high spatial resolution, we hypothesize that the processing of EFE could be influenced by high-level cortical areas (i.e. orbitofrontal and somatosensory cortices) or subcortical structures through a rapid activation of the amygdala (Mermillod et al., 2009, 2010; McFadyen et al., 2017) which would go on to modify perceptual processing of the expression. Among neural models that support the influence of top-down factors on perceptual processing (Niedenthal et al., 2010), most of them suggest that perceptual recognition in temporal cortical areas could be influenced by top-down information generated in the orbitofrontal cortex during the recognition of emotions (Kveraga et al., 2007; Barrett and Bar, 2009). In those models, early neural activity provided by frontal areas would allow a prediction that guides further bottom-up visual processes. Neuroimaging studies with high spatial resolution will have to determine the possible involvement of frontal cortical areas as the origin of the top-down effects reported in the current article.

Another potential limitation of the current research lies in the sample size and participants' characteristics. This population was composed of Bachelor degree students similar in age, cultural environment, gender, and level of education. This could constitute a methodological limitation and future studies investigating this research question could investigate top-down processes during recognition of emotional expressions using more diverse populations. We also acknowledge that the size of our different samples was relatively low (24; 20 and 15 participants for the behavioral, EMG and EEG study, respectively). Despite the consistency of our results across the three studies and even if these sample sizes are consistent with other studies in the field (Achaibou et al., 2008), further experiments should replicate and extend the current findings with larger numbers of participants since the power of behavioral, physiological or neurological effect could be unstable with lower sample sizes (e.g., Simmons et al., 2011; Schönbrodt and Perugini, 2013).

Finally, further studies will have to examine whether this modulation of early neural activity could be mediated by a differential allocation of attentional resources to perceptual processes (simple quantitative changes in neural processes) or whether the top-down modulation is able to qualitatively modify directly the *perception* of emotions. More precisely, our current data does not allow to determine whether the top-down effects reported here qualitatively modify the perceptual processes occurring at the level of the extrastriate cortex or whether it only constitutes a quantitative boost under the influence of attentional processes. Another important question for further research is to determine the extent to which a non-specific boost observed at the quantitative level of neural and physiological processes (i.e., independently to the congruency between social information and the EFE) is related to further qualitative evaluation observed at a subjective level (and related the congruency between the social valence and the emotion expressed by the target). In other words, it remains to be explained how the interaction between social valence and emotional expressions observed at a subjective level is related to the mere allocation of neural and physiological resources (i.e., irrespective to the social valence of the stimuli) at the early stages of the processing of emotions.

## Conclusion

To conclude, our data provide evidence that top-down social information about a target expressing an emotion modifies the perception of this emotion at subjective, physiological, and neural levels. In addition, our results suggest that the effect of social label on early neural activity does not systematically vary as a function of its valence (either positive or negative) and the EFE, but, alternatively, produces a global increase of early neural (EEG) and later peripheral (EMG) activity compared to the perception of emotional expressions presented without social context. These findings support and extend recent models of embodied condition, suggesting that the neural and physiological processing of emotional expressions could be modulated by the social context since its very first perceptual stages (Niedenthal et al., 2010; Wang and Hamilton, 2012). However, further studies should determine whether the effect we obtained with a social label is specific to social information or if other types of relevant and semantic information, emotional or non-emotional, could produce similar top-down influences.

## Ethics statement

This study was carried out in accordance with the recommendations of the “Comité de Protection des Personnes (CPP) Sud-Est 1” with written informed consent from all subjects. All subjects gave written informed consent in accordance with the Declaration of Helsinki. The protocol was approved by the “Comité de Protection des Personnes (CPP) Sud-Est 1, n°2009-014056-30.

## Author contributions

MM designed the study, interpreted the data and wrote the manuscript. LP-L conducted the behavioral and EEG experiment. LP-L and SH analyzed the EEG data. BB conducted the EMG experiment and analyzed the data. All authors participated to the interpretation of the results, the redaction of the article and approved the final version submitted for publication.

### Conflict of interest statement

The authors declare that the research was conducted in the absence of any commercial or financial relationships that could be construed as a potential conflict of interest.

## Appendix

**Table:**

	**Positive social label**	**Negative social label**
Male characters	Cheerful sportsman	Pedophile therapist
	Caring father	Sadistic killer
	Nice nurse	Serial rapist
Female characters	Loving mother	Asocial necrophilia
	Humanitarian doctor	Brutal coach
	Passionate teacher	Dangerous schizophrenic

## References

[B1] Achaibou A. Pourtois G. Schwartz S. Vuilleumier P. (2008). Simultaneous recording of EEG and facial muscle reactions during spontaneous emotional mimicry. Neuropsychologia 46, 1104–1113. 10.1016/j.neuropsychologia.2007.10.019 18068737

[B2] Allison T. Puce A. Spencer D. D. McCarthy G. (1999). Electrophysiological studies of human face perception. I: Potentials generated in occipitotemporal cortex by face and non-face stimuli. Cereb. Cortex 9, 415–430. 10.1093/cercor/9.5.415 10450888

[B3] Bar M. (2004). Visual objects in context. Nat. Rev. Neurosci. 5, 619–629. 10.1038/nrn1476 15263892

[B4] Barrett L. F. Bar M. (2009). See it with feeling: affective predictions during object perception. Philos. Trans. R. Soc. B Biol. Sci. 364, 1325–1334. 10.1098/rstb.2008.0312 19528014 PMC2666711

[B5] Barsalou L. W. (1999). Perceptual symbol systems. Behav. Brain Sci. 22, 577–660. 10.1017/S0140525X99002149 11301525

[B6] Beffara B. Bret A. G. Vermeulen N. Mermillod M. (2016). Resting high frequency heart rate variability selectively predicts cooperative behavior. Physiol. Behav. 164, 417–428. 10.1016/j.physbeh.2016.06.011 27343804

[B7] Beffara B. Ouellet M. Vermeulen N. Basu A. Morisseau T. Mermillod M. (2012). Enhanced embodied response following ambiguous emotional processing. Cogn. Process. 13, 103–106. 10.1007/s10339-012-0468-6 22802035

[B8] Benson P. J. Perrett D. I. (1993). Extracting prototypical facial images from exemplars. Perception 22, 257–262. 10.1068/p220257 8316513

[B9] Bentin S. Allison T. Puce A. Perez E. McCarthy G. (1996). Electrophysiological studies of face perception in humans. J. Cogn. Neurosci. 8, 551–565. 10.1162/jocn.1996.8.6.551 20740065 PMC2927138

[B10] Bublatzky F. Gerdes A. B. White A. J. Riemer M. Alpers G. W. (2014). Social and emotional relevance in face processing: happy faces of future interaction partners enhance the late positive potential. Front. Hum. Neurosci. 8:493. 10.3389/fnhum.2014.00493 25076881 PMC4100576

[B11] Calder A. J. Keane J. Cole J. Campbell R. Young A. W. (2000). Facial expression recognition by people with Mobius syndrome. Cogn. Neuropsychol. 17, 73–87. 10.1080/026432900380490 20945172

[B12] Campanella S. Falbo L. Rossignol M. Grynberg D. Balconi M. Verbanck P.. (2012). Sex differences on emotional processing are modulated by subclinical levels of alexithymia and depression: a preliminary assessment using event-related potentials. Psychiatry Res. 197, 145–153. 10.1016/j.psychres.2011.12.026 22397916

[B13] Clark V. P. Hillyard S. A. (1996). Spatial selective attention affects early extrastriate but not striate components of the visual evoked potential. J. Cogn. Neurosci. 8, 387–402. 23961943 10.1162/jocn.1996.8.5.387

[B14] Cloutier J. Ambady N. Meagher T. Gabrieli J. D. (2012). The neural substrates of person perception: spontaneous use of financial and moral status knowledge. Neuropsychologia 50, 2371–2376. 10.1016/j.neuropsychologia.2012.06.010 22732489

[B15] Dimberg U. (1990). Facial electromyography and emotional reactions. Psychophysiology 27, 481–494. 2274612 10.1111/j.1469-8986.1990.tb01962.x

[B16] Drwecki B. B. Moore C. F. Ward S. E. Prkachin K. M. (2011). Reducing racial disparities in pain treatment: the role of empathy and perspective-taking. Pain 152, 1001–1006. 10.1016/j.pain.2010.12.005 21277087

[B17] Ekman P. Friesen W. V. (1976). Pictures of Facial Affect. Palo Alto, CA: Consulting Psychologists Press.

[B18] Fridlund A. J. Cacioppo J. T. (1986). Guidelines for human electromyographic research. Psychophysiology 23, 567–589. 3809364 10.1111/j.1469-8986.1986.tb00676.x

[B19] Grèzes J. Philip L. Chadwick M. Dezecache G. Soussignan R. Conty L. (2013). Self-relevance appraisal influences facial reactions to emotional body expressions. PLoS ONE 8:e55885. 10.1371/journal.pone.0055885 23405230 PMC3566069

[B20] Havas D. A. Glenberg A. M. Gutowski K. A. Lucarelli M. J. Davidson R. J. (2010). Cosmetic use of botulinum toxin-A affects processing of emotional language. Psychol. Sci. 21, 895–900. 10.1177/0956797610374742 20548056 PMC3070188

[B21] Hess U. Adams R. B. Jr. Kleck R. E. (2007). When two do the same, it might not mean the same: the perception of emotional expressions shown by men and women, in Group Dynamics and Emotional Expression, eds Hess U. Philippot P. (New York, NY: Cambridge University Press), 33–50.

[B22] Hess U. Banse R. Kappas A. (1995). The intensity of facial expression is determined by underlying affective state and social situation. J. Pers. Soc. Psychol. 69, 280–288.

[B23] Kever A. Grynberg D. Eeckhout C. Mermillod M. Fantini C. Vermeulen N. (2015). The body language: the spontaneous influence of congruent bodily arousal on the awareness of emotional words. J. Exp. Psychol. Hum. Percept. Perform. 41, 582–589. 10.1037/xhp0000055 25915069

[B24] Kubota J. T. Ito T. A. (2007). Multiple cues in social perception: the time course of processing race and facial expression. J. Exp. Soc. Psychol. 43, 738–752. 10.1016/j.jesp.2006.10.023 17940587 PMC2031842

[B25] Künecke J. Hildebrandt A. Recio G. Sommer W. Wilhelm O. (2014). Facial EMG responses to emotional expressions are related to emotion perception ability. PLoS ONE 9:e84053. 10.1371/journal.pone.0084053 24489647 PMC3904816

[B26] Kveraga K. Ghuman A. S. Bar M. (2007). Top-down predictions in the cognitive brain. Brain Cogn. 65, 145–168. 10.1016/j.bandc.2007.06.007 17923222 PMC2099308

[B27] Lamm C. Batson C. D. Decety J. (2007). The neural substrate of human empathy: effects of perspective-taking and cognitive appraisal. J. Cogn. Neurosci. 19, 42–58. 10.1162/jocn.2007.19.1.42 17214562

[B28] Likowski K. U. Mühlberger A. Seibt B. Pauli P. Weyers P. (2008). Modulation of facial mimicry by attitudes. J. Exp. Soc. Psychol. 44, 1065–1072. 10.1016/j.jesp.2007.10.007

[B29] McFadyen J. Mermillod M. Mattingley J. B. Halász V. Garrido M. I. (2017). A Rapid subcortical amygdala route for faces irrespective of spatial frequency and emotion. J. Neurosci. 37, 3864–3874. 10.1523/JNEUROSCI.3525-16.2017 28283563 PMC6596715

[B30] Mermillod M. Droit-Volet S. Devaux D. Schaefer A. Vermeulen N. (2010). Are coarse scales sufficient for fast detection of visual threat? Psychol. Sci. 21, 1429–1437. 10.1177/0956797610381503 20817781

[B31] Mermillod M. Vuilleumier P. Peyrin C. Alleysson D. Marendaz C. (2009). The importance of low spatial frequency information for recognising fearful facial expressions. Conn. Sci. 21, 75–83. 10.1080/09540090802213974

[B32] Mondillon L. Niedenthal P. M. Gil S. Droit-Volet S. (2007). Imitation of in-group versus out-group members' facial expressions of anger: A test with a time perception task. Soc. Neurosci. 2, 223–237. 10.1080/17470910701376894 18633817

[B33] Niedenthal P. M. (2007). Embodying emotion. Science 316, 1002–1005. 10.1126/science.1136930 17510358

[B34] Niedenthal P. M. Mermillod M. Maringer M. Hess U. (2010). The Simulation of Smiles (SIMS) Model: embodied simulation and the meaning of facial expression. Behav. Brain Sci. 33, 464–480. 10.1017/S0140525X10002748 21211115

[B35] Niedenthal P. M. Winkielman P. Mondillon L. Vermeulen N. (2009). Embodiment of emotional concepts: evidence from EMG measures. J. Pers. Soc. Psychol. 96, 1120–1136. 10.1037/a0015574 19469591

[B36] Pitcher D. Garrido L. Walsh V. Duchaine B. C. (2008). Transcranial magnetic stimulation disrupts the perception and embodiment of facial expressions. J. Neurosci. 28, 8929–8933. 10.1523/JNEUROSCI.1450-08.2008 18768686 PMC6670866

[B37] Pourtois G. Dan E. S. Grandjean D. Sander D. Vuilleumier P. (2005). Enhanced extrastriate visual response to bandpass spatial frequency filtered fearful faces: time course and topographic evoked-potentials mapping. Hum. Brain Mapp. 26, 65–79. 10.1002/hbm.20130 15954123 PMC6871777

[B38] Pourtois G. Grandjean D. Sander D. Vuilleumier P. (2004). Electrophysiological correlates of rapid spatial orienting towards fearful faces. Cereb. Cortex 14, 619–633. 10.1093/cercor/bhh023 15054077

[B39] Quétard B. Quinton J. C. Colomb M. Pezzulo G. Barca L. Izaute M.. (2015). Combined effects of expectations and visual uncertainty upon detection and identification of a target in the fog. Cogn. Process. 16, 343–348. 10.1007/s10339-015-0673-1 26209302

[B40] Quétard B. Quinton J. C. Mermillod M. Barca L. Pezzulo G. Colomb M.. (2016). Differential effects of visual uncertainty and contextual guidance on perceptual decisions: evidence from eye and mouse tracking in visual search. J. Vis. 16, 28–28. 10.1167/16.11.28 27690168

[B41] Rottenberg J. Kasch K. L. Gross J. J. Gotlib I. H. (2002). Sadness and amusement reactivity differentially predict concurrent and prospective functioning in major depressive disorder. Emotion 2:135. 10.1037/1528-3542.2.2.135 12899187

[B42] Rudrauf D. David O. Lachaux J. P. Kovach C. K. Martinerie J. Renault B.. (2008). Rapid interactions between the ventral visual stream and emotion-related structures rely on a twopathway architecture. J. Neurosci. 28, 2793–2803. 10.1523/JNEUROSCI.3476-07.2008 18337409 PMC6670659

[B43] Scherer K. R. (1997). The role of culture in emotion-antecedent appraisal. J. Pers. Soc. Psychol. 73, 902–922.

[B44] Schönbrodt F. D. Perugini M. (2013). At what sample size do correlations stabilize? J. Res. Pers. 47, 609–612. 10.1016/j.jrp.2013.05.009

[B45] Schupp H. T. Flaisch T. Stockburger J. Junghöfer M. (2006). Emotion and attention: event-related brain potential studies. Prog. Brain Res. 156, 123–143. 10.1016/S0079-6123(06)56002-9 17015073

[B46] Schwartz G. E. Brown S. L. Ahern G. L. (1980). Facial muscle patterning and subjective experience during affective imagery: sex differences. Psychophysiology 17, 75–82. 7355191 10.1111/j.1469-8986.1980.tb02463.x

[B47] Schyns P. G. Goldstone R. L. Thibaut J. P. (1998). The development of features in object concepts. Behav. Brain Sci. 21, 1–54. 10097010 10.1017/s0140525x98000107

[B48] Simmons J. P. Nelson L. D. Simonsohn U. (2011). False-positive psychology: undisclosed flexibility in data collection and analysis allows presenting anything as significant. Psychol. Sci. 22, 1359–1366. 10.1177/0956797611417632 22006061

[B49] Singer T. Kiebel S. J. Winston J. S. Dolan R. J. Frith C. D. (2004). Brain responses to the acquired moral status of faces. Neuron 41, 653–662. 10.1016/S0896-6273(04)00014-5 14980212

[B50] Sloan D. M. Strauss M. E. Quirk S. W. Sajatovic M. (1997). Subjective and expressive emotional responses in depression. J. Affect. Disord. 46, 135–141. 9479617 10.1016/s0165-0327(97)00097-9

[B51] Stel M. Vonk R. (2010). Mimicry in social interaction: benefits for mimickers, mimickees, and their interaction. Br. J. Psychol. 101, 311–323. 10.1348/000712609X465424 19646328

[B52] Tortosa M. I. Lupiáñez J. Ruz M. (2013). Race, emotion and trust: an ERP study. Brain Res. 1494, 44–55. 10.1016/j.brainres.2012.11.037 23220554

[B53] Vermeulen N. Mermillod M. Godefroid J. Corneille O. (2009). Unintended embodiment of concepts into percepts: sensory activation boosts attention for same-modality concepts in the attentional blink paradigm. Cognition 112, 467–472. 10.1016/j.cognition.2009.06.003 19576578

[B54] Villepoux A. Vermeulen N. Niedenthal P. Mermillod M. (2015). Evidence of fast and automatic gender bias in affective priming. J. Cogn. Psychol. 27, 301–309. 10.1080/20445911.2014.1000919

[B55] Vrtička P. Andersson F. Sander D. Vuilleumier P. (2009). Memory for friends or foes: the social context of past encounters with faces modulates their subsequent neural traces in the brain. Soc. Neurosci. 4, 384–401. 10.1080/17470910902941793 19637101

[B56] Wang Y. Hamilton A. F. (2012). Social top-down response modulation (STORM): a model of the control of mimicry in social interaction. Front. Hum. Neurosci. 6:153. 10.3389/fnhum.2012.00153 22675295 PMC3366585

[B57] Winston J. S. Strange B. A. O'Doherty J. Dolan R. J. (2002). Automatic and intentional brain responses during evaluation of trustworthiness of faces. Nat. Neurosci. 5, 277–283. 10.1038/nn816 11850635

